# Application and analysis of the arthroscopic remnant-preserving technique to rotator cuff tear: a retrospective study

**DOI:** 10.3389/fsurg.2025.1602709

**Published:** 2025-06-30

**Authors:** Tao Bao, Yuxia Yang, Yangyang Hu, Wenyong Fei

**Affiliations:** ^1^ Department of Orthopedics, Chun'an County First People's Hospital, Hangzhou, China; ^2^ Department of Orthopedics and Sports Medicine, Northern Jiangsu People's Hospital Affiliated to Yangzhou University, Yangzhou, China

**Keywords:** remnant tissue on the footprint area, shoulder arthroscopy, remnant-preservation technique, remnant-removal technique, rotator cuff injuries

## Abstract

**Background:**

Few studies have investigated the effect of remnant tissue in the footprint area on rotator cuff repair. This study aimed to compare the clinical and structural outcomes of remnant-preservation and remnant-removal techniques during arthroscopic rotator cuff repair.

**Methods:**

This study compared arthroscopic remnant-preservation (RP) and remnant-removal (RR) techniques for rotator cuff repair in 68 patients (2–5 cm tears) with footprint remnant tissue. Patients were divided into the RP group (*n* = 33) and the RR (*n* = 35) group. Outcomes were assessed preoperatively and at 3, 6, and 24 months postoperatively. Evaluations included visual analog scale (VAS) pain scores, American Shoulder and Elbow Surgeons (ASES) and Constant–Murley (CS) scores, shoulder mobility, and MRI-based healing (Sugaya grade).

**Results:**

At 3 months, the RP group reported lower VAS scores than those in the RR group (1.4 ± 0.6 vs. 1.8 ± 0.7, *P* = 0.017). Shoulder forward flexion and abduction improved significantly in the RP group at 6 months (flexion, 159.5 ± 1.7° vs. 151.2 ± 1.7°; abduction, 145.1 ± 10.9° vs. 137.2 ± 11.1°, *P* ≤ 0.005) and 24 months (flexion, 167.2 ± 1.9° vs. 161.1 ± 1.8°; abduction, 161.2 ± 8.4° vs. 153.2 ± 13.9°, *P* ≤ 0.025). ASES scores were higher in the RP group at 6 months (95.0 ± 4.8 vs. 91.4 ± 6.8, *P* = 0.014) and 24 months (94.9 ± 3.8 vs. 89.4 ± 6.9, *P* = 0.001). MRI at 24 months demonstrated superior rotator cuff healing in the RP group (*P* = 0.008).

**Conclusions:**

The remnant-preservation technique was associated with reduced early postoperative pain, better functional recovery in shoulder mobility, and enhanced rotator cuff healing compared with remnant removal. These findings suggest that preserving footprint remnant tissue during arthroscopic repair may optimize clinical and structural outcomes.

## Introduction

Rotator cuff injuries are a common type of shoulder injury (1), mainly in acute trauma to the shoulder and in chronic degeneration of the rotator cuff (2). The prevalence of rotator cuff injuries in the general population is estimated to be 22.1% (3). When a rotator cuff tear occurs, the torn rotator cuff tendon usually detaches from the footprint area on the greater tuberosity of the humerus (4). However, in some cases, the rotator cuff tear is located proximal to the bone–tendon interface in the footprint area, and some rotator cuff tendinous tissue can remain in the footprint area. Footprint area remnant tissue could occur in up to 75% of acute rotator cuff injuries under ultrasound (5).

In arthroscopic suturing of the footprint with remnant rotator cuff injuries, the conventional surgery aims to remove the remnant of the rotator cuff from the footprint and visualize the suture anchors at the humeral greater tuberosity at the footprint. However, this is at the expense of the remnant rotator cuff tissue in the footprint. It has been shown that the rotator cuff tissue at the footprint contains many mechanoreceptors, stem cells, and microvascular blood supply (6–8). The arthroscopic remnant-preserving technique for rotator cuff surgery in the footprint area has rarely been reported in arthroscopic surgery. By preserving the natural remnant rotator cuff tissue in the footprint area, the proximal end of the torn rotator cuff is sutured and fixed to the remnant tendon in the footprint area, thus preserving the natural fibrocartilage layer and tendon in the rotator cuff footprint area, protecting the native structure of the rotator cuff footprint area, and allowing tendon–bone healing to be converted to tendon–tendon healing. Therefore, we hypothesize that preserving remnant rotator cuff tissue at the footprint area may enhance postoperative functional recovery and tendon healing.

The objective of this study was to compare the remnant-preservation (RP) technique and remnant-removal (RR) technique in the treatment of remnant tendon tissue on the footprint area in rotator cuff tear under arthroscopy. We postulated that the remnant-preserving technique was associated with a better clinical outcome than the remnant-removal technique for remnant tissue on the footprint area in the rotator cuff tear. Through our 2-year clinical retrospective study, it is found that the remnant-preserving technique can obtain better flexion and abduction of the shoulder joint, obviously relieve early shoulder pain after the operation, and achieve a stronger rotator cuff healing effect.

## Material and methods

### Case inclusion criteria

This is a single-center retrospective study. Between May 2020 and November 2021, we recruited 68 patients with residual tissue in the plantar area and randomly divided them into two groups. Patients underwent arthroscopic repair through remnant-preserving and remnant-removal techniques, performed by one senior orthopedic surgeon. Patients who underwent surgery from May 2020 to December 2020 received the remnant-removal technique, and patients who underwent surgery from January 2021 to November 2021 received the remnant-preserving technique. All patients were divided into two groups according to the mode of operation: RP group or RR group. In the RP group, the remnant tissue of the footprint area was preserved for repair of the rotator cuff with a suture bridge. In the RR group, the remnant tissue of the footprint area was removed for repair of the rotator cuff with a suture bridge.

We included patients who met the following criteria: (1) preoperative MRI and intraoperative arthroscopic diagnosis of a full rotator cuff tear with remnant rotator cuff tissue over the footprint area; (2) weakness in abduction of the affected shoulder; (3) pain in the affected shoulder; (4) no neurovascular injury and no proximal humerus related fractures; (5) no contraindications to general anesthesia; and (6) good compliance. We excluded patients with (1) a history of previous surgery on the shoulder; (2) a history of shoulder instability, superior labrum anterior and posterior injury (SLAP), and Bankart injury; (3) muscle atrophy due to nerve injury in the shoulder; (4) combination of other major diseases that cannot tolerate surgery; or (5) a history of previous surgery, closure, and small needle treatment on the shoulder.

**Figure 1:**
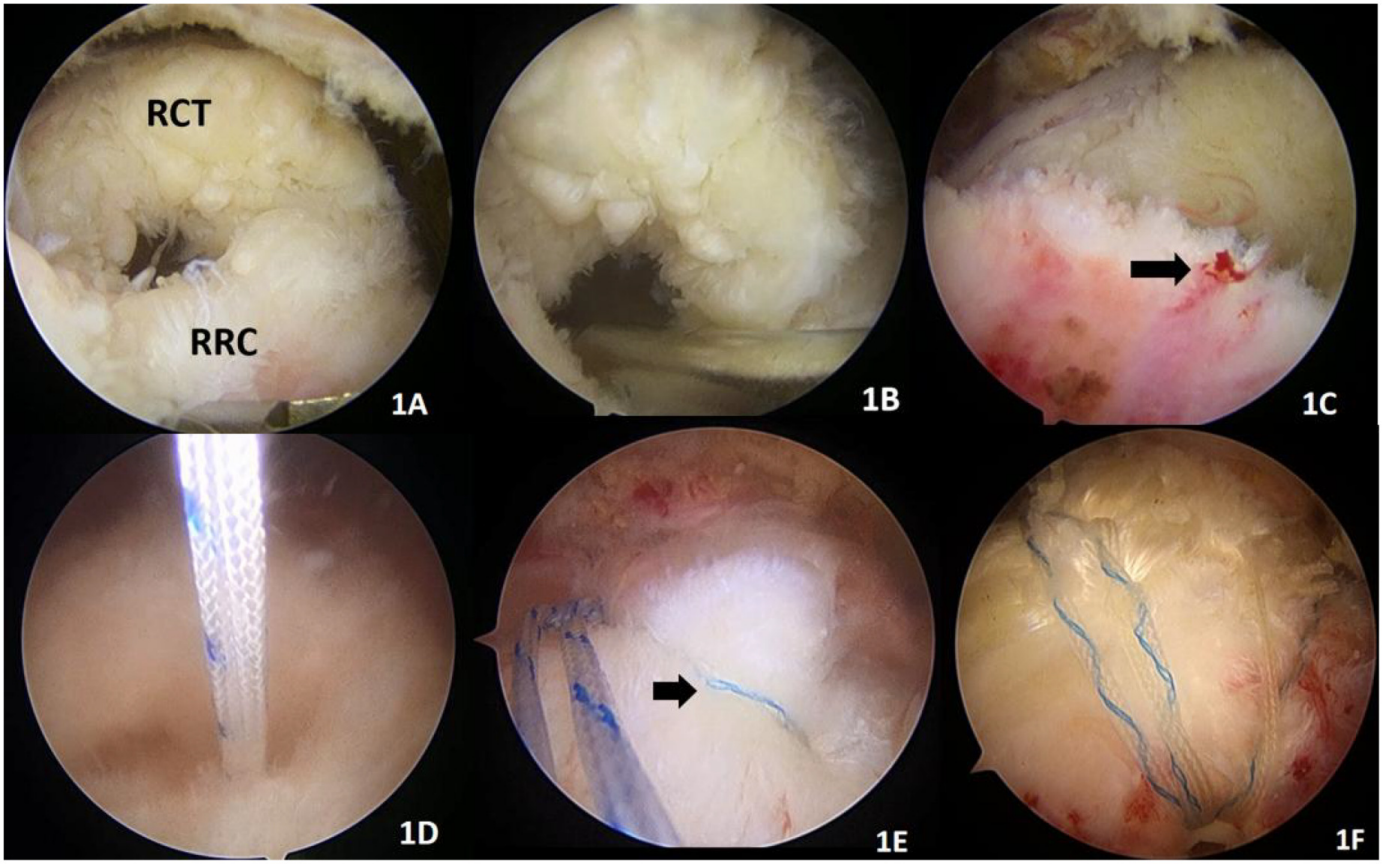
Remnant-preserving technique. **(A)** RCT (proximal rotator cuff tear), RRC (remnant rotator cuff in the footprint area). **(B)** We fresh the remnant rotator cuff with a shaver. **(C)** The black arrow indicates a bleeding spot on the remnant rotator cuff, indicating a blood supply to the remnant rotator cuff. **(D)** The remnant is preserved, and a sutured internal row anchor staple (4.5 mm absorbable, Smith & Nephew, USA) is placed in the footprint area. **(E)** The remnant and proximal end of the rotator cuff are sutured “edge to edge” as indicated by the black arrow. **(F)** Repair with two internal (4.5 mm absorbable, Smith & Nephew, USA) plus two external PushLock (4.5 × 24 mm PushLock, Arthrex, USA) suture bridges. Suturing of the proximal end and remnant of the rotator cuff tear.

**Figure 2:**
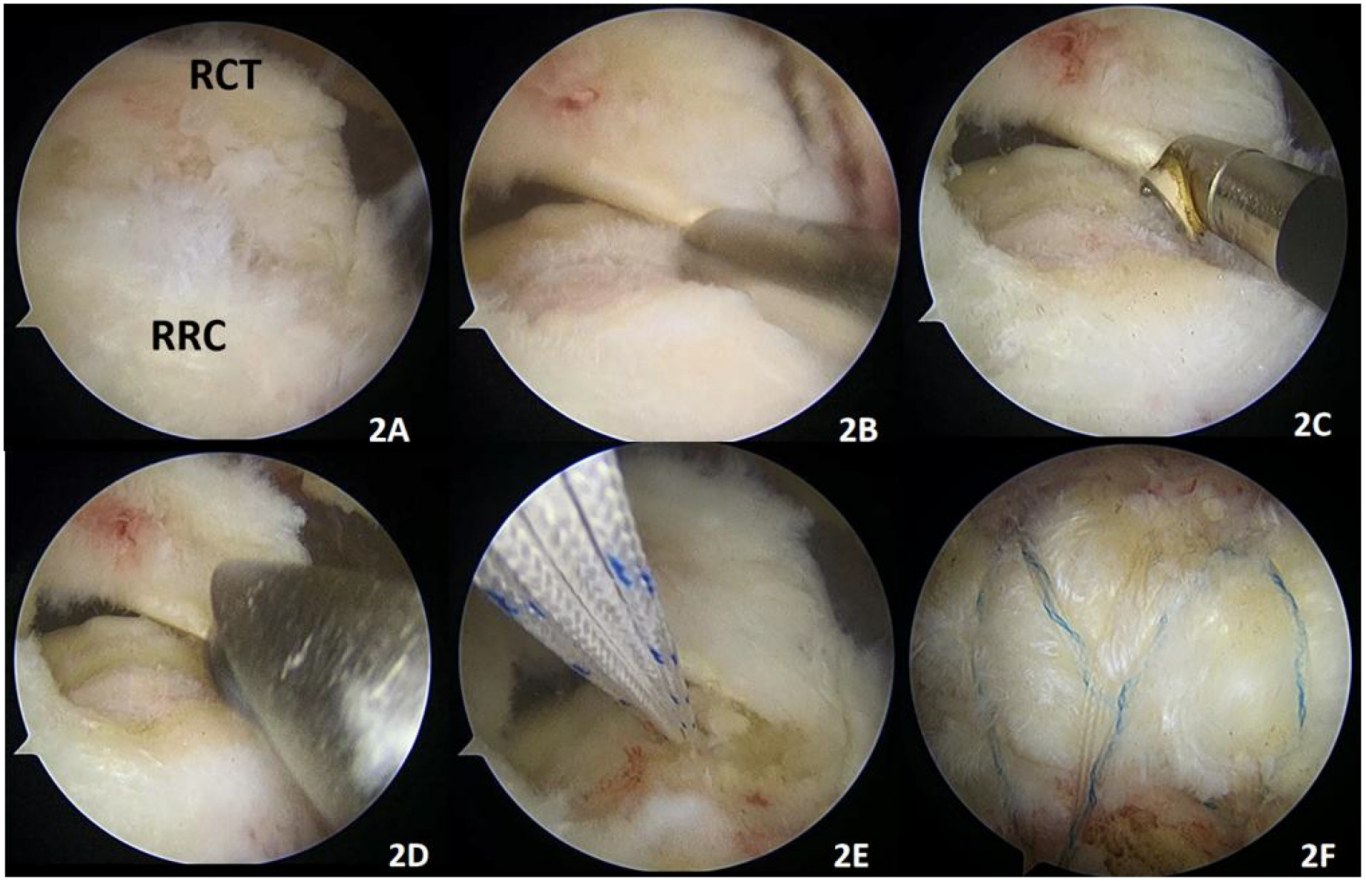
Remnant-removal technique. **(A)** RCT (proximal rotator cuff tear), RRC (remnant rotator cuff in the footprint area). **(B)** Removal of the remnant rotator cuff with a shaver. **(C)** Partial debridement of the remnant tissue with a radiofrequency tip. **(D)** Debridement of the humeral footprint area with a grinding drill. **(E)** Implantation of two internal anchors (4.5 mm absorbable, Smith & Nephew, USA) in the footprint area. **(F)** Implantation of two PushLock (Arthrex, USA, 4.5 × 24 mm diameter) to be used as a suture bridge to repair the rotator cuff.

**Figure 3:**
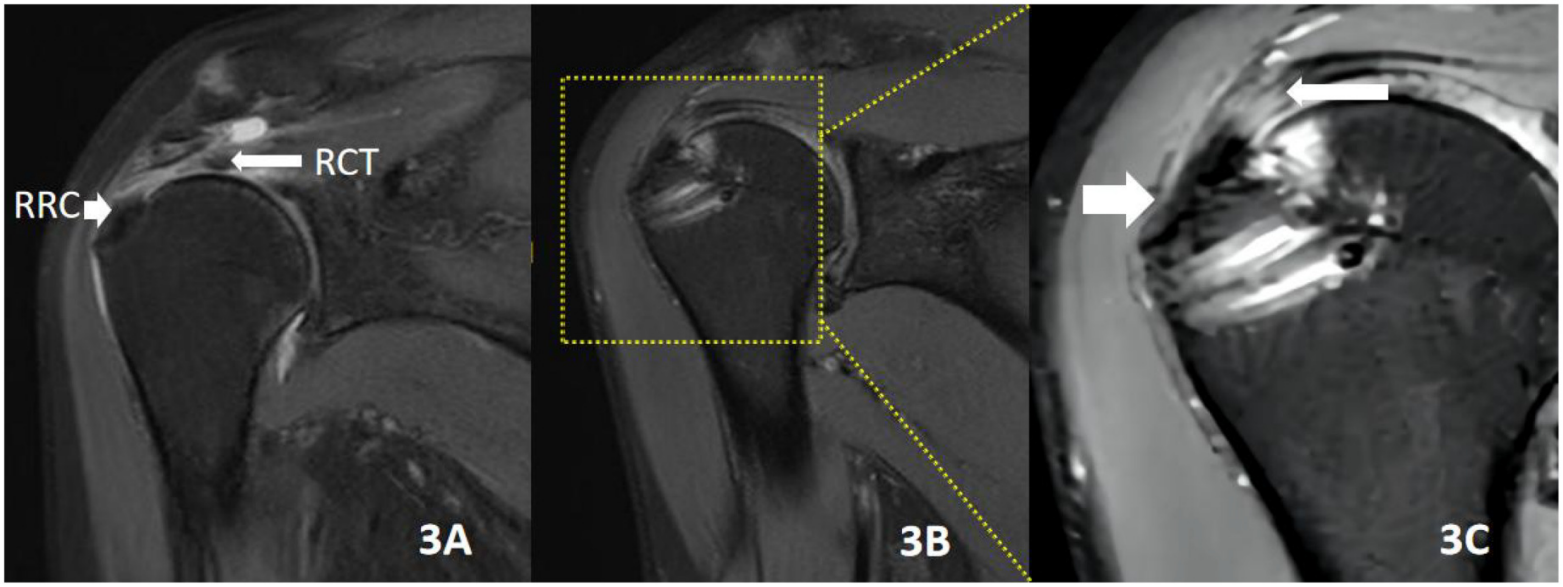
Male patient, 55 years old, traumatic injury causing shoulder pain and discomfort for 4 months, undergoing remnant-preserving techniques repair. **(A)** RCT (proximal rotator cuff tear), RRC (remnant rotator cuff in the footprint area). **(B)** MRI of the shoulder joint repeated 2 years after surgery, with good rotator cuff healing. **(C)** A magnified 3× image in the yellow box in **B**. The short white arrow on the left indicates the preservation of the original rotator cuff migration structure in the rotator cuff and footprint area after the remnant-preserving technique. The long white arrow on the right indicates the tendon–tendon healing formed in the torn portion of the rotator cuff.

**Figure 4:**
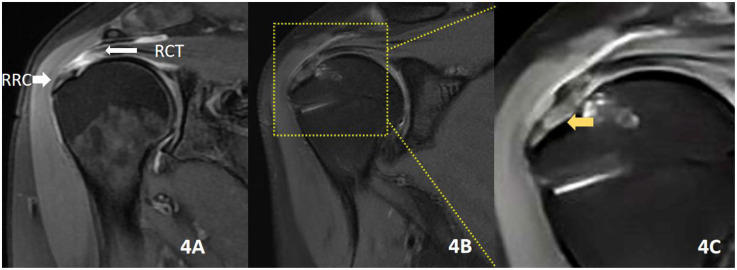
Male patient, 53 years old, with a traumatic injury causing shoulder pain and discomfort for 3 months, who underwent remnant-removal technique repair. **(A)** RCT (proximal rotator cuff tear), RRC (remnant rotator cuff in the footprint area). **(B)** MRI of the shoulder joint repeated 2 years after surgery. **(C)** Shows **B** 3× magnified image in the yellow box; the yellow arrow indicates the repair after the RR technique. The tendon–bone healing interface is scar healing without the original tissue of the rotator cuff migration area.

Remnant tendon tissue on the footprint area in the rotator cuff tear was diagnosed by preoperative MRI and confirmed during arthroscopic surgery (Figures 1A, 2A, 3A, 4A).

### Surgical technique

General anesthesia with tracheal intubation, lateral recumbency, and traction of the upper limb on the affected side at 45° of abduction in the shoulder skin retractor was performed by the same surgeon which lasted for 90–120 min. Intraoperatively, the anesthetist controlled the systolic blood pressure at 100–110 mmHg (1 mmHg = 0.133 kPa). The glenohumeral joint was inserted through a standard posterior approach to observe the long head of the biceps, glenoid labrum, articular cartilage, and rotator cuff injury and to further release the anterior bundle of the middle and inferior glenohumeral ligaments and rotator cuff space under the scope. Subacromial space using the posterior approach, lateral approach, posterior–lateral approach, and subacromial decompression was routinely completed in patients with subacromial impingement syndrome. The size of the rotator cuff tear was observed and measured. The proximal superior aspect of the rotator cuff with more retraction was released with a radiofrequency tip, and the torn proximal rotator cuff tissue was freshly debrided with a shaver.

In the RP group, the rotator cuff tissue with remnant tendon at the footprint area was partially freshly shaved. Care was taken to protect the remnant rotator cuff tissue at the footprint area and avoid excessive shaving. The use of a radiofrequency tip to clean the remnant tendon in the footprint area was avoided. To balance the preservation of the remnant tendon and freshness and maximize the healing rate, the depth of freshness of the remnant tendon at the footprint area was limited to 2 mm.

Soft tissue gripper to reset the retracted rotator cuff tissue toward the footprint area, implanting one to two anchors with wires (4.5 mm absorbable, Smith & Nephew, USA) in the inner row at the footprint area of the rotator cuff stop. If there were more remnant tendon tissue in the rotator cuff footprint area that affected the visual field, the arthroscopic lens would be inserted from the posterior approach into the glenohumeral joint to confirm the direction and depth of anchor staple placement. If there was a lot of remnant rotator cuff tissue, the most marginal suture would be placed “edge to edge” on the proximal and remnant tissue of the footprint. One or two external staples (4.5 × 24 mm PushLock, Arthrex, USA) were further used to press the tendon on the lateral aspect of the greater tuberosity. Thus, the proximal end of the torn rotator cuff was tightly attached to the remnant tendon tissue (Figure 1).

In the RR group, the remnant tendon tissue in the footprint area was thoroughly removed with a shaver or radiofrequency tip. The bone cortex of the humeral footprint area was polished to the cancellous bone with a high-speed abrasive drill. Similarly, one or two anchors (4.5 mm absorbable, Smith & Nephew, USA) with sutures were implanted at the footprint area of the rotator cuff, the proximal rotator cuff tissue was closed with mattress sutures, the trailing suture was knotted, and one or two external rows of staples (4.5 × 24 mm PushLock, Arthrex, USA) were used to complete the suture bridge. Thus, the torn proximal rotator cuff overlaid the cancellous bone of the greater tuberosity (Figure 2).

**Figure 5:**
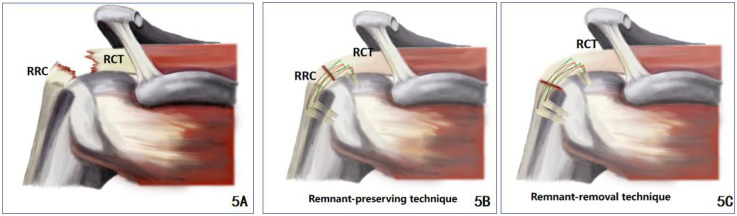
**(A)** A schematic diagram of a rotator cuff tear with a remnant rotator cuff in the footprint area, with the RCT showing the proximal rotator cuff tear and the RRC showing the remnant rotator cuff in the footprint area; **(B)** RR technique repair; **(C)** remnant-preserving techniques repair.

The specific surgical operation was schematically illustrated in Figure 5.

### Postoperative rehabilitation

After surgery, the shoulder was protected by a 45° shoulder abduction pack. Within 3 weeks after surgery, functional exercises for elbow flexion and extension were carried out under the guidance of the rehabilitation doctor, and 3–4 weeks after surgery, passive functional exercises for forward flexion and external rotation were started. Active functional exercises of the shoulder should be started 8 weeks after surgery. After 3 months, resistance exercises and daily activities of the shoulder joint should be possible. After 6 months, shoulder weight-bearing labor and physical activities can be performed.

### Evaluation of clinical and MRI assessment outcomes

All patients were reviewed at the specialist clinic postoperatively, and visual analog scale (VAS), American Shoulder and Elbow Surgeons (ASES), Constant–Murley (CS) shoulder score, shoulder forward flexion, abduction, neutral external rotation, and internal rotation range of motion were recorded at 3, 6, and 24 months postoperatively to assess postoperative shoulder function. The range of shoulder forward flexion, abduction, and neutral external rotation of the shoulder joint was measured by an angle measuring instrument, and the value was the maximum angle of shoulder joint movement of patients. To facilitate statistical analysis, according to the CS score, internal rotation score was redefined: put the hand behind the body, thumb upright, thumb reached thigh (0 point), hip (1 point), sacroiliac joint (2 points), L5 vertebral level (3 points), L4 vertebral level (4 points), L3 vertebral level (5 points), L2 vertebral level (6 points), L1 vertebral level (7 points), T12 vertebral level (8 points), T9–11 vertebral level (9 points), and the level of the scapula (10 points).

At 24 months postoperatively, scapular plane abduction muscle strength was measured using the direct tension scale method proposed by Cools et al. (9). The abduction muscle strength (in pounds) of the affected and healthy shoulder was measured separately by the physician using the same tension scale (accuracy, 0.01 pounds), and the ratio of the affected to the healthy side abduction muscle strength (affected side/healthy side) was used as the assessment method. At the final follow-up, the efficacy of the rotator cuff repair was assessed using an MRI review of the shoulder according to the Sugaya (10) staging: Type I, with normal thickness and a continuous low signal at all levels; Type II, normal thickness and complete continuity but localized high signal; Type III, <1/2 normal thickness but complete continuity; Type IV, 1–2 levels in both the oblique coronal and oblique sagittal positions; and Type IV, small discontinuities at 1–2 levels in both the oblique coronal and oblique sagittal positions, suggesting a small total tear. Sugaya Types IV and V are defined as rotator cuff re-tears.

### Statistical analysis

All statistical analyses were performed using SPSS version 25.0 (SPSS Inc., Chicago, IL, USA), and the level of significance was set at *P* < 0.05. The Shapiro–Wilk method was used to test the normality of the data. Measures that conformed to a normal distribution (age, tear size, size of remnant tissue, BMI, duration of disease, VAS score, ASES score, CS score, shoulder range of motion data) were expressed as (mean ± standard deviation), and the data were compared using independent sample data *t*-test. As the data (shoulder range of motion data and ASES, CS, and VAS scores) were repeatedly measured, the overall data were compared using the Friedman test.

The distribution of Sugaya grades at the final follow-up was compared between groups using the Mann–Whitney *U*-test; count data (sex, side, diabetes, smoking) were expressed as number of cases, and the *χ*^2^ test was used for comparison between groups.

## Results

### Patient demographic characteristics

A total of 68 patients were included, 33 in the RP group and 35 in the RR group. All patients were followed up for 2 years. There was no significant difference in demographic characteristics between the two groups (Table 1).

**Table 1:** Demographic data.

Variables	RP group (*n* = 33)	RR group (*n* = 35)	*P*-value
Age (mean ± SD), year	61.0 ± 9.0	60.1 ± 7.8	0.645
Sex: female/male, *n*	20/13	20/15	0.965
Side of involvement: left/right, *n*	15/18	16/19	1.000
Symptom duration, month	5.6 ± 3.7	5.1 ± 3.8	0.567
Size of remnant tissue, cm	3.1 ± 0.8	3.4 ± 0.7	0.112
Rotator cuff tear size, cm	3.7 ± 0.9	3.9 ± 0.9	0.312
History of trauma: yes/no, *n*	18/15	19/16	1.000
BMI	21.4 ± 2.8	21.4 ± 2.7	0.917
History of diabetes: yes/no, *n*	3/30	4/31	0.751
Smoking history: yes/no, *n*	7/26	9/26	0.662

The statistical data (gender and side) are expressed as percentages, and the χ^2^ test was used for comparison between groups, with *P* < 0.05 being a statistically significant difference.

### Results of clinical functional assessment

At 3, 6, and 24 months postoperatively, the total postoperative scoring system showed a significant improvement in the RP and RR groups. Data for both groups at the same follow-up time points were shown in Tables 2–4.

**Table 2:** Shoulder range of motion data.

Variables	Time	RP group (*n* = 33)	RR group (*n* = 35)	*P-*value
Forward flexion (°)	Preoperative	137.4 ± 5.4	122.7 ± 5.2	0.056
	3 months postoperative	157.4 ± 1.9	156.4 ± 1.9	0.719
	6 months postoperative	159.5 ± 1.7	151.2 ± 1.7	0.001**
	24 months postoperative	167.2 ± 1.9*	161.1 ± 1.8*	0.025**
Abduction (°)	Preoperative	112.2 ± 21.7	109.2 ± 27.3	0.586
	3 months postoperative	142.1 ± 14.5	137.0 ± 18.5	0.212
	6 months postoperative	145.1 ± 10.9	137.2 ± 11.1	0.005**
	24 months postoperative	161.2 ± 8.4*	153.2 ± 13.9*	0.006**
External rotation (°)	Preoperative	50.3 ± 17.4	47.7 ± 14.5	0.507
	3 months postoperative	46.9 ± 10.8	48.12 ± 10.4	0.651
	6 months postoperative	48.7 ± 8.3	49.5 ± 7.8	0.691
	24 months postoperative	55.4 ± 7.2*	53.5 ± 7.3*	0.290
Internal rotation (point)	Preoperative	5.6 ± 3.2	5.3 ± 2.6	0.714
	3 months postoperative	6.6 ± 1.3	6.7 ± 1.6	0.653
	6 months postoperative	6.5 ± 0.9	6.5 ± 1.0	0.895
	24 months postoperative	9.4 ± 0.7*	9.1 ± 0.9*	0.139

* Indicates a statistical difference between the last follow-up and preoperative (*P* < 0.05).

** Statistically significant.

**Table 3:** VAS score.

Variables	RP group (*n* = 33)	RR group (*n* = 35)	*P*-value
Preoperative	4.1 ± 1.4	4.6 ± 1.5	0.149
3 months postoperative	1.4 ± 0.6	1.8 ± 0.7	0.017*
6 months postoperative	1.0 ± 0.6	1.1 ± 0.7	0.321
24 months postoperative	0.9 ± 0.6	1.1 ± 0.6	0.122
*P*-value	0.001	0.001	

VAS, visual analog scale.

* Statistically significant.

**Table 4:** ASES and CS scores.

Variables	Time	RP group (*n* = 33)	RR group (*n* = 35)	*P*-value
ASES score	Preoperative	53.5 ± 13.3	52.1 ± 17.6	0.714
	3 months postoperative	74.0 ± 6.2	77.2 ± 8.4	0.075
	6 months postoperative	95.0 ± 4.8	91.4 ± 6.8	0.014*
	24 months postoperative	94.9 ± 3.8	89.4 ± 6.9	0.001*
*P*-value		0.001	0.001	
CS score	Preoperative	55.0 ± 15.1	50.6 ± 18.2	0.290
	3 months postoperative	67.9 ± 4.1	67.4 ± 8.5	0.757
	6 months postoperative	86.8 ± 6.5	85.9 ± 5.2	0.542
	24 months postoperative	86.5 ± 7.3	83.6 ± 5.2	0.063
*P*-value		0.001	0.001	

ASES, American Shoulder and Elbow Surgeons; CS, Constant–Murley shoulder.

* Statistically significant.

In the comparison of mobility between the two groups, there were no statistically significant differences in forward flexion, abduction, neutral external rotation, or body-side internal rotation before surgery, at 3, 6, and 24 months after surgery (*P* > 0.05 for all comparisons). At 6 and 24 months postoperatively, the differences in forward flexion (159.5 ± 1.7° and 167.2 ± 1.9° in the RP group and 151.2 ± 1.7° and 161.1 ± 1.8° in the RR group) and abduction (145.1 ± 10.9° and 161.2 ± 8.4° in the RP group and 137.2 ± 11.1° and 153.2 ± 13.9° in the RR group) were statistically significant (*P* < 0.05). There was no statistically significant difference in shoulder mobility between the two groups at other postoperative time periods (*P* > 0.05) (Table 2).

The VAS score at 3 months postoperatively was lower in the RP group (1.4 ± 0.6) than in the RR group (1.8 ± 0.7) points (*P* < 0.05), and there was no statistically significant difference between the preoperative and 6 and 24 months postoperatively VAS scores in both groups (*P* > 0.05) (Table 3).

In terms of functional scores, the difference in ASES scores between the two groups at 6 and 24 months postoperatively was statistically significant (*P* < 0.05), and there was no statistically significant difference in ASES scores between preoperative and 3 months postoperatively (*P* > 0.05). There was no statistically significant difference (*P* > 0.05) in CS scores preoperatively and 3, 6, and 24 months postoperatively (Table 4).

### Imaging outcomes at the last follow-up

MRI results showed that the Sugaya class classification in the RP group was 78.8% (26/33) in Type I, 15.2% (5/33) in Type II, and 6.0% (2/33) in Type III. The Sugaya class classification in the RR group was 48.6% (17/35) in Type I, 28.6% (10/35) in Type II, 20.0% (7/35) in Type III, and 2.8% (1/35) in Type IV. The difference in Sugaya grade classification for postoperative healing of the rotator cuff between the conserving and disabling groups was statistically significant (*P* < 0.05). There was no statistically significant difference in the rate of rotator cuff re-tear at 24 months postoperatively (RP = 0/33; RR = 1/35) between the two groups.

## Discussion

In our study, with a follow-up of >2 years, we found that the rotator cuff remnant-preserving technique was superior to the remnant-removal technique in terms of early postoperative pain relief, shoulder mobility, and rotator cuff healing.

The normal anatomy of the rotator cuff has four zones, including tendon, uncalcified fibrocartilage, mineralized fibrocartilage, and then bony structures (11, 12), which ensure smooth mechanical stress transfer between tendon and bone. The main purpose of all rotator cuff repair surgery is to restore this native structure of the rotator cuff itself as much as possible, to withstand the motion load required for shoulder joint movement, thereby obtaining better shoulder joint function (13, 14). The current clinical practice for rotator cuff tears with remnant tendon tissue on the footprint is to remove the remnant tendon tissue from the footprint area, expose and debride the footprint area of the greater tuberosity of the humerus, and then reconstruct the footprint area. The disadvantage of this method of repair is that the postoperative healing interface between tendon and bone is scar healing and there is no natural fibrocartilage layer (15). This scar healing does not have the histological characteristics of the normal bone tendon, and the connection is not strong enough, resulting in a high rate of re-tears after rotator cuff suturing (16–20). Therefore, how to surgically repair rotator cuff injuries to achieve optimal healing has been a hot topic of discussion and exploration.

The remnant-preserving technique reduces the removal of remnant rotator cuff tissue and preserves more of the footprint rotator cuff mechanoreceptors, which helps to better maintain shoulder proprioception and therefore achieve better shoulder mobility. Previous research has identified numerous mechanoreceptors within the rotator cuff tissue that are involved in the proprioception of the shoulder joint (8, 21), which transmits information about shoulder movement, position, and forces exerted to the central nervous system for better control of the fine movements of the shoulder joint (22–25). The remnant-preserving technique preserves more of the footprint rotator cuff mechanoreceptors, which reduces pain associated with uncoordinated shoulder movements. It has been shown that shoulder joint pain and poor healing after rotator cuff repair will result in reduced postoperative shoulder mobility and poorer ASES scores (26–28). In this study, comparing the shoulder mobility and ASES scores of the two groups of patients at the 6-month postoperative and 24-month postoperative follow-up, the RP group showed better forward flexion and abduction mobility, and the ASES scores of the RP group were significantly higher than those of the RR group. This further demonstrates that the residual retention technique better maintains proprioception of the shoulder joint and results in a more pronounced functional recovery of the shoulder joint.

The remnant-preserving technique can relieve early postoperative shoulder pain. This technique reduces surgical manipulation of the footprint area remnant tendon removal and humeral decortication and reduces bone edema in the humeral footprint area, thus eliminating the need for excessive vascular remodeling of the rotator cuff tear, further reducing the release of inflammatory factors and helping to relieve postoperative shoulder pain. It has been shown that the peak onset of pain after rotator cuff suturing is 6–8 weeks postoperatively and may be related to intra-tendon angiogenesis (29), remodeling, and the release of inflammatory factors during the healing period of the rotator cuff suture (30, 31). In this study, there was a statistical difference in the VAS scores between the RP and RR group at 3 months after surgery. Patients in the RP group showed more significant pain reduction than those in the RR group, further suggesting that the remnant-preserving technique was effective in reducing early postoperative pain after rotator cuff surgery.

The remnant-preserving technique further maintains the native structure of the rotator cuff footprint (from tendon, uncalcified fibrocartilage, mineralized fibrocartilage to bony structures) by preserving the natural rotator cuff remnant tissue in the footprint area. This allows smooth mechanical stress to transfer between tendon to bone, resulting in a stronger rotator cuff to grand tuberosity healing. Using the remnant-preserving technique, anatomical repair of transtendinous rotator cuff tears can be achieved without excessive tension, and a biomechanical condition that promotes satisfactory tendon healing is established owing to the preserved remnant tissue (32). Some animal studies have also reported beneficial results of this treatment with preserved remnant tissue (33–35). Aoki et al. (33) found in dogs that attaching the severed tendon end to the remaining tendon end healed better than attaching it to the fibrocartilage layer. In the rabbit model, Sun et al. (34) reported that the entire natural attachment structure on the rotator cuff footprint was preserved and the superior-to-inferior tendon–bone connection in conventional rotator cuff repair was replaced with a tendon–tendon connection, which was superior to the tendon–bone connection in both biomechanical and histological assessments. Similarly, Su et al. (35) found, in a rabbit model, that a stronger rotator cuff-large nodal failure tension could be obtained in the group with the preserved remnant tissue than in the group with the removed remnant tissue. Studies have reported a positive correlation between the degree of healing after rotator cuff suturing and patients’ postoperative shoulder pain and mobility (36). In our study, there was a statistically significant difference between the two groups in shoulder ASES scores at 6 and 24 months postoperatively, with the RP group having a more dominant shoulder mobility capacity. A comparison of MRI of the two groups at the 24-month postoperative (see Figures 3, 4) shows that the RP group had a primitive rotator cuff footprint migration zone at the tendon–bone interface, whereas the RR group formed scar tissue at the tendon–bone interface. These clinical results demonstrate that the technique of preserving the residual tendon results in a stronger and more pristine healing of the rotator cuff to the greater tuberosity. Better shoulder mobility and function can be achieved postoperatively.

Remnant-preserving technique by preserving the remnant tissue in the footprint area can protect more of the rotator cuff blood supply and promote rotator cuff healing. Remnant-preserving technique repair procedure when freshening up the remnant tissue in the footprint area, bleeding spots can be seen on the rotator cuff in the footprint area (Figure 1C), further demonstrating the presence of local tissue blood flow. The posterior rotator humeral artery is the main vessel supplying the supraspinatus and infraspinatus tendons (7, 37). The posterior rotator humeral artery supplies blood to the rotator cuff so that it flows from the humeral attachment to the proximal rotator cuff. Some studies have found that there is still a good blood supply to the remnant tissue over the footprint area after rotator cuff tears (38, 39).

In addition, the remnant-preserving technique can provide a better source of autologous stem cells for rotator cuff healing (40). Huang et al. (6) obtained qualified 52 remnant rotator cuff tissues from the footprint area in 154 patients with rotator cuff tears. Tendon-derived stem cells (TDSCs) capable of differentiating into fibrochondrocytes could be isolated from remnant tissues. Nagura et al. (41) also isolated TDSCs from healthy or ruptured rotator cuff tissues. Tsai et al. (42) also obtained rotator cuff tissue from five patients with rotator cuff tears (men aged 34, 40, and 60 years and women aged 45 and 56 years) and isolated multipotent mesenchymal stem cells (MSCs) from them, which can have a good myogenic differentiation potential. These studies all confirm that the remnant tissues in the footprint area were active tissues containing stem cells that can differentiate into fibrochondrocytes and myogenic cells. Therefore, the remnant-preserving technique may provide better stem cell conditions for rotator cuff to large nodule healing, resulting in better healing outcomes. In this study, there was a statistically significant difference in the Sugaya grading on MRI review at 24 months postoperative between the two groups. The final follow-up MRI review Sugaya grading was significantly better in the RP group, further suggesting that better rotator cuff healing could be achieved by the remnant-preserving technique.

This study confirmed that the remnant-preserving technique can achieve better clinical outcomes than the remnant-removal technique, which had implications for a large number of rotator cuff surgeries in clinical practice. This study had several limitations. First, this is a retrospective study based on a single center with a relatively small sample size. Thus, the findings in this study may need to be further validated in future multicenter large studies. Second, the follow-up period of the patients was short, and the long-term differences between the two groups need further investigation to inform clinical practice.

## Conclusions

In conclusion, for patients with rotator cuff injury with a footprint remnant tissue, the remnant-preserving technique can achieve better shoulder flexion and abduction mobility, a more obvious reduction of early postoperative shoulder pain. The remnant-preserving technique may achieve a stronger rotator cuff healing effect by protecting the original migrating structures, blood supply, and stem cells of the remnant rotator cuff tissue, which is worth promoting in clinical practice. In the future, the remnant-preserving technique combined with the bone marrow technique can possibly achieve better clinical results in the treatment of the rotator cuff.

## Data Availability

The raw data supporting the conclusions of this article will be made available by the authors, without undue reservation.
